# Determinants of Arsenicosis Patients’ Perception and Social Implications of Arsenic Poisoning through Groundwater in Bangladesh

**DOI:** 10.3390/ijerph7103644

**Published:** 2010-10-14

**Authors:** M. Mizanur Rahman Sarker

**Affiliations:** Department of Economic Systems Analysis, Institute of Economic Research, Graduate School of Economics, Hitotsubashi University, 2-1 Naka, Kunitachi, Tokyo 186-8603, Japan; E-Mail: ed084002@g.hit-u.ac.jp; or mrs_bd@yahoo.com; Tel.: +81-42-580-8360; Fax: +81-42-580-8360

**Keywords:** logit model, social implication, perception, arsenicosis, Bangladesh

## Abstract

Adverse human health effects ranging from skin lesions to internal cancers as well as widespread social and psychological problems caused by arsenic contaminated drinking water in Bangladesh may be the biggest arsenic calamity in the world. From an arsenicosis patients survey, this paper empirically analyzes the determinants of arsenicosis patients’ perception about chronic arsenic poisoning and social and psychological implications of arsenicosis. In this study, cross-sectional data were collected from the Matlab and Hajiganj Upzillas of Chandpur district which are known to be highly contaminated with arsenic in their underground water. Respondents informed that arsenic poisoning causes a wide range of social and psychological problems. Female respondents were less vulnerable in the case of social problems (p < 0.01) and more vulnerable for the psychological problems (p < 0.001) of arsenicosis than male respondents. The results based on logit analysis showed that education (p < 0.01) and household income (p < 0.05) were significantly correlated to respondents’ perception about arsenicosis. The arsenicosis related special program (s) needs a clear understanding of people’s perception about arsenic exposure for abating the health burden as well as social and psychological problems.

## 1. Introduction

Arsenic contamination of drinking water is one of the great concerns for public health throughout the world. According to the International Agency for Research on Cancer [1] around 100 million people in the world, including about 13 million in the United States, are chronically exposed to inorganic arsenic. Although >20 countries have been affected by arsenic contamination of drinking water, the situation is perhaps the most devastating in Bangladesh because of the number of affected people [2]. Nationwide, 6,528 people will die from cancer every year and a total of 326,400 people in a period of 50 years and around 2.5 million people will develop keratoses, hyperpigmentation, cough, chest sounds, breathlessness, weakness glucosuria and high blood pressure over that period [3]. Systematic survey throughout the country by the Department of Public Health Engineering (DPHE) and the British Geological Survey (BGS) have estimated that nearly 21 million people of about 47 districts are drinking arsenic contaminated ground water [4] which is well above the standard limit of 0.05 mg/L of Bangladesh (the WHO guide line value is 0.01 mg/L [5]). Securing arsenic-free ground-water has been technically problematic in many parts of Bangladesh [6–8]. A variety of state agencies and NGOs are searching for optimum mitigation strategies, in order to reduce the risk from drinking poisoned groundwater [9].

Arsenic patients may not feel ill or look ill, other than some skin pigment discoloration, but they are stripped of their status in society and adopt a virtual identity as ‘dangerous’ people [10]. Chronic arsenic exposure is associated with many human health conditions, including skin lesions and cancers of the liver, lung, bladder and skin [11–13] as well as other noncancer health effects such as adverse reproductive outcomes, neurological disorders and impaired cognitive development in children [14–17]. Arsenic contamination has a profound impact at both the individual and community levels. Reports have attributed disease and death caused by arsenic toxicity to lack of knowledge about the source of this metal [18]. Fear of contagiousness has separated families, created social isolation in schools and led to avoidance of people living in highly contaminated regions [19]. Therefore, it is important to identify the determinants of the patients’ perception about arsenicosis and to examine the gender differences of social and psychological sufferings from chronic arsenic poisoning for reducing the disease burden and avoidable deaths. The present study explores the arsenicosis patients’ perception and problems by examining four specific questions. First, what is the socio-economic conditions of arsenicosis sufferers? Second, what is the major problems of arsenicosis sufferers in case of child development, getting marriage and married life? Third, do substaintial gender differences exist in the perception of social and psychologial implications of arsenocosis? Finally, how do the different determinants associate with arsenicosis patients’ perception?

## 2. Material and Methods

### 2.1. Study Area

Two Upazilas namely Hajiganj and Matlab out of seven Upazilas of Chandpur district were selected as they are known to be highly contaminated with arsenic in the underground water and located in southern region of Bangladesh. The upazila is the second lowest tier of administrative government in Bangladesh. The districts of Bangladesh are divided into sub-districts called Upazilas. At present, there are 482 upazilas in Bangladesh.The British Geological Survey [4] reported that there is high prevalence of arsenic in tube well water in southeast and southern Bangladesh. The melanosis, leucomalanosis and keratosis symptoms were identified around 11%, 2% and 2% of the respondents, respectively in the Hajiganj [20]. It is characterized by densely populated area and agrarian economy producing principally rice, wheat, vegetables, jute, fish, milk and poultry.

### 2.2. Data Collection

The study is based on primary data. Field survey, interview, communication and interaction with different stakeholders were conducted for primary data collection. A three-stage sampling procedure was undertaken. Firstly, two Upazilas were selected purposively. Secondly, preliminary information about the patients has been collected from the department of public health engineering, local NGOs personnel, health workers, family planning workers, extension personnel and ICDDR, B field workers, and thirdly, then 150 (90 female and 60 male) respondents were selected from 458 patients for the present study. Sample respondents were selected by using simple random sampling frame work. The female respondents were more than male respondents because the response rate was higher for female respondents than male respondents. The response rate of respondents was 88%. A pre-design pre-tested interviewer made questionnaire was used to conduct the survey. Two trained interviewers (one man and one woman) conducted the survey by face-to-face interview. Attention was given to the wording of the questions during questionnaire design, so that the respondents found it simple and could understand it easily.

### 2.3. Variables

The socio-economic variables include age, sex, family size, house hold income, occupation, source of drinking and cooking water. Family members were categorized into adult male, adult female and children (less than 12 years). The highest level of educational attainment was categorized into three levels, namely illiterate/no education, below Secondary School Certificate (SSC), and Higher Secondary Certificate. House-hold income was calculated based on respondents’ self report. Respondents were asked to classify their job into one of five different occupations, namely housewife, agriculture, business, service, and others. The source of water was classified into five categories; tube well, pond, rainwater harvest, river and canal. Social implications of the chronic arsenic poisoning from drinking ground water was evaluated by asking the question “Do you think that the arsenicosis is a cause of dislike of other people to participate of social activities?” The knowledge of respondents regarding the psychological implications of the chronic arsenic poisoning from drinking ground water was evaluated by asking indirect question. To assess the psychological implications of arsenicosis, the respondents were asked the question “Do you think that the arsenicosis is an evil spirit/God’s curse/sin/contagious disease?” Perception regarding the chronic arsenic poisoning from drinking ground water was evaluated by asking whether the respondent had a clear perception of adverse health effects of arsenic. The respondents were asked the question “Do you think that the drinking of arsenic-contaminated water is a cause of health problems?” If the answerer was ‘yes’, then again the respondents were asked the question about arsenic related diseases, “Do you know that the arsenic-contaminated water is a cause of Melanosis, Leucomelanosis, Keratosis, Hyperkeratosis?” Those who know the arsenic-contaminated water is a cause of above mentioned diseases, the respondents consider as they have clear perception about adverse health effect of arsenic-contaminated drinking water. The dependent variable was valued at one if the respondent has clear perception about adverse health effect of arsenic-contaminated drinking water and zero if he/she has no clear perception.

### 2.4. Statistical Analyses

This study used descriptive statistics to describe the data and logit regression model to explore the determinants of the respondents’ perception about arsenic exposure to human health. All analyses were performed by using SPSS package.

#### 2.4.1. Descriptive Statistics

Descriptive analyses involved calculations of frequency distribution, percentage, mean and tabular statistics for reporting the socioeconomic characteristics, skin lesion, social and psychological implications of arsenicosis. Chi-square test was used to find out the association between social and psychological implication of arsenicosis and gender of respondents.

#### 2.4.2. Logit Regression

Logit regression is used for prediction of the probability of occurrence of an event. Logit regression allows one to predict a discrete outcome from a set of variables that may be continuous, discrete, dichotomous, or a mix of any of these. Generally, the dependent or response variable is dichotomous, such as presence/absence or success/failure of an event. To determine respondents’ perception about arsenicosis the following Logit model was fitted to the empirical data which is given by Gujarati [21]:

$$L_{i}=\text{ln}\left(\frac{P_{i}}{1-P_{i}}\right)=\beta_{0}+\beta_{1}X_{1i}+\beta_{2}X_{2i}+\beta_{3}X_{3i}+\beta_{4}X_{4i}+\beta_{5}X_{5i}+\beta_{6}X_{6i}+U_{i}$$

where:

L_i_ = 1 if the respondent has clear perception about arsenicosis and 0 otherwise
P_i_ = the probability of the respondents’ clear perception about arsenicosis
X_1_ = educational qualification of the arsenicosis patient (year of schooling)
X_2_ = household income of the arsenicosis patient (in Tk. per year)
X_3_ = age (in years)
X_4_ = 1 for male respondent
X_4_ = 0 for female respondent
X_5_ = 1 for married respondent
X_5_ = 0 for unmarried respondent
X_6_ = duration of suffering from arsenicosis (in years)
β_1_ to β _6_ are coefficients of the respective explanatory variables.

## 3. Results

### 3.1. Socio-Demographic Background

The socioeconomic and demographic characteristics of surveyed arsenicosis patients are presented in Table 1. Respondents comprised 60 males and 90 females. The average age of the respondent was 50 ± 19 years. Mean age ± SD of male and female were 54 ± 15 and 47 ± 21 years, respectively. The average number of adult male, adult female and children per family were found to be 2.34, 2.52 and 1.12, respectively. About 52% of respondents had no formal education but 46% respondents had primary and 2% of respondents had secondary and higher secondary education. Agriculture was the main source of income and the average monthly income of respondent household was 3,874 Tk. (US$ 56.97) per month. The major occupation of respondents were 57%, 22%, 8%, 6% and 7% for housewife, agriculture, business, service, and others, respectively. Respondents reported that the main source of drinking water was tube well. On the other hand, respondents in the study area stated that the majority (74%) used pond water as a major source for cooking purposes.

### 3.2. Social Implication of Arsenicosis

Attempts were made to learn of the feelings of the arsenic patients regarding participation in their social activities. A considerable portion of the respondents (32%) reported that their neighbors dislike the arsenic patients’ participation in their social activities.

#### 3.2.1. Gender Wise Distribution of Perception of Social Implications

Table 2 examines whether men or women were better informed about the social implications of arsenicosis. Among the total respondents 37% reported that arsenic exposure had social implications and 63% reported that arsenicosis had no social implications. When examined against gender it was found that more males (57%) thought that arsenicosis had a social implication than females (24%).

#### 3.2.2. Arsenic Poisoning and Child Development

Physical and mental development of a child in an arsenic-affected family may be interrupted because of different reasons. A considerable percentage of respondents (28%) assumed that unhappy family life may hamper psychological development of the children. The reported reasons for this were as follows: the child might be deprived of love and affection due to arsenic-related problems of the father and/or mother; children might be neglected socially due to parent’s unhealthy condition; father’s and/or mother’s illness may result in increased workload and create physical and mental pressure on the children.

#### 3.2.3. Arsenicosis Problems for Seeking Marriage

Victims may face a crisis in maintaining their usual emotion, love and affection within their daily life. Women with arsenicosis usually suffer the most in this regard. A majority of the respondents (64%) informed that a girl might face difficulties in getting married due to arsenicosis. The reasons mentioned for facing difficulties by the girls were as follows: girls look unattractive and less glamorous due to arsenic poisoning (41%); nobody likes to get married to a girl patient (8%); and a newly married arsenic-affected girl may act as transmitting superstition (4%). According to 12 per cent of the respondents, men having arsenicosis were not feeling confident in getting married and eight per cent of the respondents thought that men who had signs of arsenic exposure to the body were discouraged by their friends, relatives and neighbors in getting married. Findings show that men became disinterested in marrying a girl who had signs of arsenicosis due to various reasons, e.g., such a girl would cause unhappy family condition; such a girl would be sexually malfunctioned; arsenic causes considerable physical damage to a girl; additional money will be required for treatment of a newly married woman.

#### 3.2.4. Problems in Married Life

About one third of the respondents (32%) mentioned that married couples who were suffering from arsenic poisoning may face unhappy conjugal or family life. Findings show that an unhappy conjugal situation might arise from the following reasons: anxiety and possibility of ending a marriage, physical disability due to arsenicosis. About one fourth of the respondents (27%) indicated that a marriage might end in divorce if the wife suffered from arsenicosis. The anticipated reasons were as follows: arsenic problem of a married women may deteriorate the conjugal relationship with her husband (11%); the husband may lose attraction to his wife because of his wife’s deteriorating physical appearance (4%); the husband's fear of getting infected from his wife (2%); and superstition (6%).

### 3.3. Perception of Psychological Implications of Arsenicosis

Table 3 shows the relationship of gender with the perception of psychological implications of arsenicosis. The distribution of the respondents regarding psychological implications varied substantially between the sexes. It is evident that males (53%) were less conversant than females (91%) with the psychological implication of arsenicosis.

### 3.4. Logit Analysis for the Respondents’ Perception about Arsenicosis

Logit model was applied to determine the respondents’ perception about arsenicosis. In this model the explanatory variables were education of respondent, household income, age, gender (dummy), marital status (dummy) and duration of suffering from arsenicosis of respondents. The results of a logit equation for respondents’ perception about arsenicosis are presented in Table 4. The logit analysis showed that two variables namely education of the patient and household income, were statistically significant at 1% and 5% level, respectively.

## 4. Discussion

The present study shows that the average household size of 5.97 was slightly higher than the average 5.3 of Chandpur district [22]. The respondents were also asked the question “Who is responsible for water collection in your family?” The response was 92% women and 8% men. This is consistent with other findings. In rural Bangladesh, domestic water collection and management is predominantly undertaken by women and girls, who spend considerable amount of time and energy under various conditions on a daily basis to collect drinking water for their families [23]. It is rare for men to participate in domestic water collection [24].

Arsenic poisoning causes a wide range of health problems as well as social and psychological sufferings such as community refusal, social discrimination, unhappy conjugal life, child development problems, mental despondency etc. Findings show that non-participation in the social activities by the patients was not only the cause of self-restrained participation in the social activities but also there was dislike of other people to participate in their social activities. Some family members also do not like to talk and hesitate to come close to arsenicosis patients. Studies found that social and economic loss for people in arsenic areas were acute and rapidly worsening [25]. Arsenic-related weakness and illness causes further economic damage, as people suffering from arsenicosis were increasingly unable to work [26]. Most of the arsenicosis patients can not afford their treatment cost which leads to social crisis and distress selling [27]. Men are more active in social activities than women; this may possibly be due to cultural differences between men and women. Gender difference was statistically significant (p < 0.01). It is consistent with a previous study showing that the social implications of arsenicosis for men and women do vary [24]. Besides health effects, arsenicosis also generates problems in social and daily life and disturbs the marriage system. There are reports of broken marriages and problems in getting married. Women afflicted with skin spots or lesions (the first visible symptoms of arsenicosis) have been reported to be treated as contagious and often abandoned or denied marriage. In the same village, women/girls with visible signs of arsenicosis are facing more difficulty in getting married compared to men; increased dowry is often demanded of the woman/girl’s family [24]. Arsenicosis would not only pose a threat for getting married but it also creates many problems within married life. A study found that 8% females reported that they had been abandoned by their husbands [28]. Nearly 53% of the women identified the biggest problem to be marriageability issues and rejection of women [29]. The arsenic problem is not only a threat to physical health of victims but it would also impact on mental health of sufferers because of false beliefs that still persist, such as arsenicosis is an evil spirit or God's curse or a contagious disease. Gender difference of psychological sufferings was statistically significant (p < 0.001). Sufferings from water poisoning differ for male and female according to social status and locations [30–32].

The results indicate that with the increase in schooling years and house-hold income of the patient, the probability of the respondents’ heightened perception about arsenicosis would be greater. Socio-economic status variables were related to the knowledge of the health problems of arsenic exposure [19]. A previous study [2] found that people with higher socio-economic status (non labor occupation of the head of the household and better housing) were more aware of the health effects of arsenic. On the other hand, findings of other health surveys have shown that awareness is related to knowledge of a correct behavioral or lifestyle modification [33,34], but these studies are not associated with perception of arsenicosis.

The main strategies should be aimed at ensuring the arsenic free water, awareness raising program(s) and finally, income generating activities for rural poor people. Women should be trained to know about alternative ways to get arsenic free water. To ensure the participation of the female local leaders, especially female ward members and upazila vice-president in the awareness build up program. False belief, lack of resources and treatment facilities are major barriers in overcoming arsenicosis patients’ problems.

There were several limitations in this study. For practical reasons, 150 sample respondents were selected for analysis. This study was based on cross sectional data. The present study was not adjusted for other risk factors such as physical activity and smoking. The most of the data were collected by using self reported data which might be affected by the differential reporting behavior of men and women. It is plausible that both the occurrence of any health problems and their consequences are worse in less advantage socioeconomic groups, but we were not able to test this in our data because the size of our sample did not allow us to separate the households into more detailed income brackets.

## 5. Conclusions

The current study examined the social implications of arsenic poisoning and its seriousness. The study found that arsenicosis has negative social and psychological implications which leads to social discrimination, uncertainty, injustice, human rights violation and threats to family and conjugal life. Women were less educated and psychologically more vulnerable than men. These findings suggest that special education/training programs may need to target individuals with low income and education status in order to improve perception about consequences of chronic arsenic poisoning; this would be an important element for abating the increasing social crisis.

Ideally, political commitments and institutional policies are needed to allocate resources to ensure arsenic-free drinking water. Until then, a nation-wide large-scale study based on the current survey will be of high utility to assess the real burden of arsenicosis on various occupations, socio-economic segments, gender and age groups. In view of the increasing burden of arsenicosis this should be considered as an essential part of the national poverty alleviation and human development strategy of Bangladesh.

## Figures and Tables

**Table 1.** Socio-demographic characteristics of the surveyed arsenicosis patients.

**Characteristics**	**Values**
**Average age of the respondent (years)**	**50 ± 19**
Male	54 ±15
Female	47 ± 21

**Average family member (No)**	**5.97 ± 3.5**
Adult male	2.34 ± 2.0
Adult female	2.51 ± 1.5
Children	1.12 ± 2.5

**Literacy**	
No education	78 (52%)
Below S.S.C.	69 (46%)
S.S.C. and H.S.C.	3 (2%)

**Average monthly household income (Tk.)**	3,874 ± 3,252

**Major occupation of the respondents**	
Agriculture	33 (22%)
Business	12 (8%)
Service	9 (6%)
Housewife	85 (57%)
Others	7 (11%)

**Source of drinking water**	
Tube wells	123 (82%)
Pond	12 (8%)
River	13 (9%)
Canal	9 (6%)
Rain water	33 (22%)

**Source of cooking water**	
Tube wells	33 (22%)
Ponds	111 (74%)
Others	6 (4%)

**Responsible for water collection**	
Men	12 (8%)
Women	138 (92%)

*Note*: Number of respondents in parentheses, 68 Tk. = 1 $US

**Table 2.** Distribution of respondents by gender and their perception on any social implication of arsenic exposure.

**Sex of respondent**	**Social implication of arsenicosis**		
	**Yes**	**No**	**Total**
Female	22 (24%)	68 (76%)	90 (100%)
Male	34 (57%)	26 (43%)	60 (100%)
Total	56 (37%)	94 (63%)	150 (100%)

**Table 3.** Distribution of respondents by gender and their perception about psychological implication of arsenicosis.

**Sex of respondent**	**Psychological implication of arsenicosis**		
	**Yes**	**No**	**Total**
Female	82 (91%)	08 (09%)	90 (100%)
Male	32 (53%)	28 (47%)	60 (100%)
Total	114 (77%)	36 (23%)	150 (100%)

**Table 4.** Results of the estimated logit equation of respondents’ perception about arsenicosis (t statistics in parentheses).

**Variables**	**Coefficients**
Education	1.907 ** (4.362)
Income	1.421 * (2.377)
Male respondent (dummy)	0.431 (1.032)
Age	0.804 (1.482)
Respondent is married (dummy)	0.031 (.603)
Symptoms present longevity	0.137 (0.321)
Intercept	−1.704 ** (9.864)

** significance at 0.01 probability level,

* significance at 0.05 probability level.
